# Risk factors for malnutrition and its impact on prognosis in patients with severe pneumonia

**DOI:** 10.3389/fnut.2026.1787876

**Published:** 2026-06-10

**Authors:** Yan-Yan Qin, Rui Jiang, Yu-Zhi Wu, Fang Liu, Chen Yang

**Affiliations:** Department of Respiratory and Critical Care Medicine, Shanxi Provincial People's Hospital, Taiyuan, Shanxi, China

**Keywords:** Cox proportional hazards model, global leadership initiative on malnutrition, logistic regression, malnutrition, prognosis, severe pneumonia

## Abstract

**Background:**

Malnutrition is common in acute and critical illness, but its clinical correlates and prognostic relevance in severe pneumonia remain incompletely characterized. This study investigated factors associated with malnutrition at admission and its association with short-term outcomes in patients with severe pneumonia.

**Methods:**

In this retrospective cohort study, adults with severe pneumonia treated between January 2021 and December 2024 were consecutively included. Malnutrition at admission was defined using the Global Leadership Initiative on Malnutrition (GLIM) framework. Univariable and multivariable logistic regression analyses were used to identify factors associated with malnutrition. Logistic regression and Cox proportional hazards models were used to examine the association between malnutrition and clinical outcomes. Pre-specified subgroup and sensitivity analyses were performed.

**Results:**

Among 211 eligible patients, 59 (28.0%) were classified as having malnutrition at admission. Malnourished patients had a less favorable admission profile, including greater comorbidity burden, more severe inflammatory and respiratory abnormalities, and higher illness severity. In multivariable analysis, lower body mass index, lower serum albumin, higher procalcitonin, higher Acute Physiology and Chronic Health Evaluation II (APACHE II) score, heart failure, and chronic kidney disease were independently associated with malnutrition. Malnutrition was also associated with less favorable short-term outcomes, including higher in-hospital and 28-day mortality, greater intensive care unit utilization, longer hospitalization, and higher hospital costs. After adjustment for major clinical confounders, malnutrition remained significantly associated with mortality-related endpoints, intensive care unit (ICU) admission, invasive mechanical ventilation, and a lower rate of discharge alive. These findings were directionally consistent in subgroup and sensitivity analyses.

**Conclusions:**

GLIM-defined malnutrition was frequent in severe pneumonia and was associated with an adverse clinical profile and less favorable short-term outcomes. These findings suggest that malnutrition may represent a clinically relevant marker of vulnerability in this population.

## 1 Introduction

Severe pneumonia remains a major driver of acute respiratory failure, intensive care utilization, and short-term mortality, and contemporary guidance emphasizes early recognition of high-risk phenotypes and timely escalation of supportive and etiologic therapies (1–3). In this context, malnutrition is clinically relevant because it frequently coexists with severe infection and critical illness, and it is biologically linked to impaired immune competence, respiratory muscle weakness, dysregulated inflammatory responses, and delayed recovery (4–6). Current critical care nutrition guidance therefore recommends systematic nutritional risk screening, objective assessment, and timely initiation of appropriate nutrition therapy as integral components of intensive care unit (ICU) management (7–9).

A persistent challenge is that disease-related malnutrition in acute care settings is heterogeneous and may be under-recognized when assessment relies on single parameters such as body mass index or serum proteins (10). The Global Leadership Initiative on Malnutrition (GLIM) framework provides a structured approach that integrates phenotypic and etiologic criteria, and a recent consensus update further refined the definition to improve consistency across clinical and research settings (11). In parallel, implementation-focused work and guideline appraisal studies highlight ongoing gaps between recommendations and routine practice, including variability in screening intensity, diagnostic thresholds, and documentation pathways (12). Hospital-based studies have shown that malnutrition identified using standardized criteria is associated with adverse clinical trajectories and greater resource utilization, further supporting its clinical relevance in acute illness (13).

For patients with severe pneumonia, nutritional risk is likely shaped by an interaction between baseline reserves, comorbidity burden, and acute disease severity, including systemic inflammation and organ dysfunction (14). Severity-stratified pneumonia guidelines explicitly recognize that host factors and physiologic derangements influence outcomes, yet the determinants of malnutrition and the extent to which malnutrition is associated with prognosis beyond established severity indices remain incompletely characterized (15). Moreover, ICU-oriented risk tools such as the modified Nutrition Risk in the Critically Ill (mNUTRIC) score, as well as practical nutritional indices used in respiratory cohorts, have been associated with short-term outcomes, underscoring the potential value of integrating nutritional status into risk stratification for severe pneumonia (16, 17). Against this background, evaluating factors associated with malnutrition and clarifying its prognostic implications in severe pneumonia may inform early identification strategies and support individualized nutritional and critical care management.

## 2 Methods

### 2.1 Study design and participants

This retrospective cohort study consecutively enrolled patients with a confirmed diagnosis of severe pneumonia who received treatment at our institution between January 2021 and December 2024. Patients were stratified into a malnutrition group and a non-malnutrition group according to the presence of malnutrition at admission, as determined by documented nutritional assessment and diagnostic criteria in the medical record. Inclusion criteria were: (1) hospitalization during the pre-defined study period; (2) diagnosis of severe pneumonia based on established clinical, laboratory, and radiologic criteria; (3) availability of baseline demographic data, comorbidity information, and key admission assessments; and (4) completion of a nutritional evaluation within the early hospitalization period sufficient to classify malnutrition status. Exclusion criteria were: (1) age <18 years; (2) pregnancy or lactation; (3) transfer to our institution after substantial prior inpatient treatment or incomplete pre-admission data preventing accurate baseline assessment; (4) hospital stay <24 h or death on arrival before completion of baseline assessment; and (5) missing or implausible key variables required for group allocation or outcome evaluation. Informed consent was obtained from all participants and/or their legal guardians. The study protocol was reviewed and approved by the hospital's ethics committee. All procedures were conducted in accordance with relevant guidelines and regulations, adhering to the ethical principles outlined in the Declaration of Helsinki. Participant confidentiality was strictly maintained, with all personal identifiers removed prior to data analysis.

### 2.2 Diagnostic criteria

Severe pneumonia was diagnosed based on compatible clinical features of lower respiratory tract infection together with new or progressive pulmonary infiltrates on chest radiography or computed tomography (CT). Severity was defined according to the American Thoracic Society/Infectious Diseases Society of America (ATS/IDSA) criteria for severe community-acquired pneumonia, requiring the presence of at least one major criterion or three or more minor criteria. Major criteria included respiratory failure requiring invasive mechanical ventilation and septic shock requiring vasopressors. Minor criteria included respiratory rate ≥30 breaths/min, ratio of arterial partial pressure of oxygen to fraction of inspired oxygen (PaO_2_/FiO_2_) ≤ 250, multilobar infiltrates, confusion/disorientation, uremia (blood urea nitrogen ≥20 mg/dl), leukopenia (white blood cell count <4.0 × 10^9^/L), thrombocytopenia (platelet count <100 × 10^9^/L), hypothermia (core temperature <36.0 °C), and hypotension requiring aggressive fluid resuscitation (18, 19). Malnutrition was diagnosed at admission using the GLIM framework, which requires the presence of at least one phenotypic criterion and one etiologic criterion. Phenotypic criteria included any of the following: (1) non-volitional weight loss (>5% within the previous 6 months or >10% beyond 6 months, when documented in the medical record or confirmed during admission history taking); (2) low body mass index (BMI) (<20 kg/m^2^ for patients <70 years or <22 kg/m^2^ for patients ≥70 years); and/or (3) reduced skeletal muscle mass. In this retrospective cohort, GLIM phenotypic adjudication was based on documented recent weight loss, admission BMI, and reduced muscle mass when objective body-composition assessment or standardized clinical documentation was available. Reduced skeletal muscle mass was considered only when supported by routine-care records, including bioelectrical impedance analysis, CT-based assessment, ultrasound, or equivalent standardized documentation. Because objective body-composition assessment was not routinely performed in all patients, its absence was not interpreted as evidence of preserved muscle mass.

Etiologic criteria included: (1) reduced food intake or assimilation; and/or (2) inflammation/disease burden. In this retrospective cohort, reduced food intake was adjudicated only when supported by documented admission nutrition assessment, physician history, or nursing records indicating one of the following: estimated oral intake ≤ 50% of usual or estimated requirements for >1 week, sustained reduction in food intake for >2 weeks, or clinically documented malabsorption when applicable. When the duration or severity of reduced intake was not explicitly documented, this criterion was not considered fulfilled. In the present study, inflammation was not assigned solely on the basis of the diagnosis of severe pneumonia. Because GLIM recognizes inflammation or disease burden as an etiologic domain but does not prescribe a single mandatory biomarker threshold for retrospective adjudication, we pre-specified an operational definition based on objective indicators routinely available in the medical record during the first 24 h of admission. This approach was used to improve reproducibility of case classification in this retrospective cohort and to avoid automatically assigning the inflammation criterion solely on the basis of a diagnosis of severe pneumonia. Rather, it was operationalized using clinical and laboratory evidence of acute systemic inflammatory burden within the first 24 h of admission, including one or more of the following: C-reactive protein >10 mg/L, procalcitonin >0.5 ng/ml, abnormal white blood cell count (>10 × 10^9^/L or <4 × 10^9^/L), septic shock requiring vasopressors, or respiratory failure requiring invasive mechanical ventilation. These indicators were used only to adjudicate the GLIM etiologic domain in combination with at least one phenotypic criterion, rather than as stand-alone evidence of malnutrition. Patients meeting at least one phenotypic criterion and at least one etiologic criterion were assigned to the malnutrition group; all others were categorized as non-malnutrition.

### 2.3 Data collection and follow-up

Clinical data were retrospectively extracted from the institutional electronic medical record system, including the hospital information system, laboratory information system, radiology archive, and ICU charting platform. Data abstraction was performed using a standardized case report form by trained reviewers, and a second reviewer cross-checked a pre-specified proportion of records to ensure consistency; discrepancies were resolved by consensus with reference to source documents. Baseline variables were defined *a priori* and collected preferentially from the first assessment after admission (typically within 24 h) to reflect the initial clinical status. Collected domains included demographics and anthropometrics (age, sex, body mass index, body weight, and smoking status), comorbidities and medical history (chronic obstructive pulmonary disease (COPD), diabetes mellitus, heart failure, chronic kidney disease, malignancy history, and other clinically relevant chronic conditions), physiologic parameters at presentation (respiratory rate, heart rate, systolic/diastolic blood pressure, peripheral oxygen saturation, and oxygenation indices such as PaO_2_/FiO_2_ when available), laboratory indices reflecting inflammation and nutritional/hematologic status (white blood cell count, C-reactive protein, procalcitonin, serum albumin, pre-albumin, hemoglobin, and lymphocyte count), and disease severity, assessed using the Acute Physiology and Chronic Health Evaluation II (APACHE II) score calculated from the worst values recorded within the first 24 h after admission or ICU entry according to standard scoring rules. Symptom duration before admission was also abstracted from initial history documentation and admission records when available. For GLIM adjudication, data on recent non-volitional weight loss, BMI, available muscle-mass assessment, and reduced food intake were abstracted from admission nutrition records, initial history documentation, physician notes, and nursing intake records when available. Imaging and microbiologic information were recorded from routine care documentation, including multilobar involvement and pleural effusion on chest imaging and pathogen evidence based on available microbiologic tests (e.g., culture, antigen/serology, or nucleic-acid testing) as documented in the medical record. Treatment-related variables included initial antimicrobial regimen, escalation or combination therapy when applicable, and organ-support measures such as vasopressor use and continuous renal replacement therapy.

Data on early nutritional management during the first 72 h after admission were extracted from physician orders, nursing flow sheets, nutrition consultation records, enteral/parenteral nutrition charts, and medication-administration records when available. The extracted variables included whether formal nutrition support was provided, route of support (oral diet/oral nutritional supplements, enteral nutrition, parenteral nutrition, or combined enteral and parenteral nutrition), time from admission to first nutrition support, and, when documented, mean daily delivered energy and protein during the first 72 h. Energy adequacy was defined as delivery of at least 70% of the estimated energy target by 72 h, and protein adequacy as delivery of at least 70% of a protein target of 1.3 g/kg/day by 72 h. When detailed quantitative intake records were unavailable, energy- and protein-delivery variables were treated as missing and summarized using available-case denominators.

Follow-up focused on short-term clinical endpoints. In-hospital outcomes were obtained directly from the index hospitalization record. Twenty-eight-day outcomes were defined from the admission date, and vital status at day 28 was ascertained by review of medical records; for patients discharged before day 28, survival status and major events were determined by review of subsequent institutional encounters and, when necessary, structured telephone follow-up with the patient or legally authorized representative. Time-to-event outcomes were operationalized as follows: for 28-day survival, patients were followed from admission to death within 28 days, with censoring at day 28 or the last verified contact; for time to discharge alive, discharge was treated as the event of interest and deaths were censored at the time of death. Resource-use outcomes (hospital length of stay, ICU length of stay, and total hospitalization costs) were extracted from administrative billing and discharge summaries using the hospital's standardized accounting records. Additional in-hospital clinical outcomes and treatment-related indicators were also recorded descriptively, including ICU admission, sepsis or septic shock, acute respiratory distress syndrome, acute kidney injury, multiple organ dysfunction syndrome, vasopressor use, continuous renal replacement therapy, antibiotic escalation, and invasive mechanical ventilation during hospitalization.

### 2.4 Statistical analysis

All statistical analyses were performed using IBM SPSS Statistics, version 29.0 (IBM Corp., Armonk, NY, USA). Continuous variables are presented as mean ± standard deviation (SD) or median (interquartile range, IQR) according to data distribution and were compared using the independent-samples t test or Mann–Whitney U test, as appropriate. Categorical variables are expressed as number (percentage) and were compared using the chi-square test or Fisher's exact test. Factors associated with malnutrition were evaluated using univariable and multivariable logistic regression analyses, and the results are reported as odds ratios (ORs) with 95% confidence intervals (CIs). The association between malnutrition and binary clinical outcomes was also assessed using logistic regression analysis. Cox proportional hazards regression models were used to analyze 28-day survival and time to discharge alive, with results presented as hazard ratios (HRs) and 95% CIs. Primary analyses used a complete-case approach for occasional missingness in non-core covariates. As a sensitivity analysis, missing non-core covariate data were additionally handled using multiple imputation with 20 imputed datasets, and the results were compared with those of the complete-case analyses. All tests were two-sided, and *P* < 0.05 was considered statistically significant.

## 3 Results

### 3.1 Patient selection and group allocation

A total of 237 consecutive patients with severe pneumonia treated at our institution between January 2021 and December 2024 were screened. After eligibility assessment, 26 patients were excluded according to the predefined criteria, including 6 with hospital stay <24 h or death on arrival before completion of baseline assessment, 5 inter-hospital transfers after substantial prior inpatient treatment or with incomplete pre-admission data that precluded accurate baseline assessment, and 15 with missing or implausible key variables required for group allocation or outcome evaluation. No patients were excluded because of age <18 years, pregnancy, or lactation. The final analytic cohort included 211 patients, of whom 59 (28.0%) were classified as having malnutrition and 152 (72.0%) as not having malnutrition at admission. Because patients with missing core variables for group allocation or primary outcome ascertainment were excluded during screening, completeness for these key variables was 100% in the analytic cohort. Occasional missingness in non-core covariates was handled using a complete-case approach in the primary analyses, with multiple imputation performed as a sensitivity analysis. Detailed distributions of GLIM phenotypic criteria and body-composition assessment are shown in Supplementary Table S1.

### 3.2 Baseline characteristics

Compared with the non-malnutrition group, patients with malnutrition were older and had lower body mass index and body weight. They also had higher frequencies of chronic obstructive pulmonary disease, diabetes mellitus, heart failure, and chronic kidney disease. At admission, the malnutrition group had worse respiratory and inflammatory profiles, including higher respiratory rate, lower peripheral oxygen saturation and PaO_2_/FiO_2_ ratio, and higher inflammatory markers, while albumin, pre-albumin, hemoglobin, and lymphocyte count were lower. APACHE II score was higher, and multilobar involvement and pleural effusion were more frequent in the malnutrition group. Presumed pathogen distribution and initial combination antibiotic therapy did not differ significantly between groups (Table 1). During early hospitalization, formal nutritional support within the first 72 h was more frequently provided to patients with malnutrition at admission. Among patients who received nutritional support, the distribution of the primary nutrition route did not differ significantly between groups, and the proportion of patients initiating support within 48 h was also comparable. Quantitative documentation of energy and protein delivery was available more often in the malnutrition group. However, among patients with available records, no significant between-group differences were observed in energy adequacy or protein adequacy by 72 h. Detailed results are presented in Supplementary Table S2.

**Table 1:** Baseline demographic, clinical, laboratory, imaging, and selected early management characteristics by nutritional status.

Variable	Malnutrition (*n* = 59)	Non-malnutrition (*n* = 152)	*P*-value
Age, years	68.3 ± 9.0	64.5 ± 10.5	0.010
Male sex, *n* (%)	31 (52.5)	94 (61.8)	0.217
Body mass index, kg/m^2^	20.1 ± 2.2	23.6 ± 2.9	<0.001
Body weight, kg	55.6 ± 9.4	65.7 ± 11.7	<0.001
Current/former smoker, *n* (%)	15 (25.4)	49 (32.2)	0.334
Chronic obstructive pulmonary disease, *n* (%)	25 (42.4)	35 (23.0)	0.005
Diabetes mellitus, *n* (%)	20 (33.9)	27 (17.8)	0.011
Heart failure, *n* (%)	21 (35.6)	12 (7.9)	<0.001
Chronic kidney disease, *n* (%)	14 (23.7)	13 (8.6)	0.003
History of malignancy, *n* (%)	3 (5.1)	9 (5.9)	1.000
Respiratory rate, breaths/min	30.2 ± 6.4	26.7 ± 5.3	<0.001
Systolic blood pressure, mmHg	119.2 ± 17.5	121.9 ± 15.7	0.301
Diastolic blood pressure, mmHg	68.0 ± 12.8	72.9 ± 11.5	0.011
Heart rate, beats/min	104.2 ± 19.7	101.8 ± 17.0	0.414
Peripheral oxygen saturation, %	88.1 ± 5.2	91.1 ± 4.3	<0.001
PaO_2_/FiO_2_ ratio, mmHg	170.6 ± 59.5	221.8 ± 68.6	<0.001
White blood cell count, × 10?/L	11.7 ± 3.9	10.2 ± 3.6	0.012
C-reactive protein, mg/L	92.6 (69.0, 130.6)	71.8 (44.6, 101.4)	0.003
Procalcitonin, ng/ml	0.8 (0.6, 1.3)	0.5 (0.3, 0.8)	<0.001
Serum albumin, g/L	30.4 ± 4.3	34.6 ± 4.4	<0.001
Serum pre-albumin, mg/L	147.5 ± 42.0	192.6 ± 52.1	<0.001
Hemoglobin, g/dl	10.8 ± 1.8	11.8 ± 1.7	<0.001
Lymphocyte count, × 10^9^/L	0.8 ± 0.4	1.0 ± 0.4	<0.001
APACHE II score	19.6 ± 5.6	16.3 ± 6.1	<0.001
Multilobar involvement, *n* (%)	42 (71.2)	81 (53.3)	0.018
Pleural effusion, *n* (%)	21 (35.6)	33 (21.7)	0.038
Presumed pathogen category, *n* (%)			
Bacterial	26 (44.1)	75 (49.3)	0.833
Viral	10 (16.9)	23 (15.1)	
Mixed	8 (13.6)	15 (9.9)	
Unknown	15 (25.4)	39 (25.7)	
Initial combination antibiotic therapy, *n* (%)	42 (71.2)	93 (61.2)	0.174

APACHE II, acute physiology and chronic health evaluation II; IQR, interquartile range; PaO_2_/FiO_2_, ratio of arterial partial pressure of oxygen to fraction of inspired oxygen.

### 3.3 Univariable and multivariable logistic regression analyses of factors associated with malnutrition

In univariable logistic regression analyses, lower body mass index, heart failure, chronic kidney disease, lower serum albumin, higher procalcitonin, and higher APACHE II score were significantly associated with malnutrition. In the multivariable logistic regression model, these associations remained statistically significant, indicating that lower body mass index and serum albumin, as well as the presence of heart failure and chronic kidney disease, higher procalcitonin, and higher APACHE II score, were independently associated with malnutrition (Table 2).

**Table 2:** Univariable and multivariable logistic regression analyses of factors associated with malnutrition.

Variable	Univariable OR (95% CI)	*P-*value	Multivariable OR (95% CI)	*P*-value
Lower body mass index (per 1 kg/m^2^ decrease)	1.82 (1.49–2.22)	<0.001	1.59 (1.28–1.96)	<0.001
Heart failure	6.39 (2.92–13.99)	<0.001	3.72 (1.50–9.24)	0.005
Chronic kidney disease	3.28 (1.48–7.28)	0.003	2.41 (1.01–5.77)	0.048
Lower serum albumin (per 1 g/L decrease)	1.22 (1.14–1.30)	<0.001	1.14 (1.05–1.22)	0.001
Higher procalcitonin (per 1 ng/ml increase)	1.60 (1.23–2.09)	<0.001	1.42 (1.08–1.87)	0.012
Higher APACHE II score (per 1-point increase)	1.12 (1.06–1.19)	<0.001	1.07 (1.01–1.14)	0.028

APACHE II, acute physiology and chronic health evaluation II; CI, confidence interval; OR, odds ratio.

Univariable logistic regression analyses were first performed to screen potential factors associated with malnutrition.

Variables with P <0.10 in the univariable analyses, together with clinically relevant covariates, were considered for inclusion in the multivariable logistic regression model.

To reduce multicollinearity and overfitting, correlated variables were not entered simultaneously, and the final model was derived using backward stepwise elimination based on the likelihood-ratio criterion.

Only variables retained in the final multivariable model are presented in this table.

### 3.4 Subgroup and interaction analyses

Pre-specified subgroup analyses stratified by age, sex, COPD status, history of malignancy, and baseline disease severity showed that the associations of lower body mass index, lower serum albumin, and higher procalcitonin with malnutrition were directionally consistent across strata. No statistically significant interactions were identified for any of these subgroup analyses (all *P* for interaction >0.05), indicating no evidence of effect modification by the pre-specified stratification factors (Table 3).

**Table 3:** Subgroup analyses and interaction tests for key predictors of malnutrition.

Stratification factor (subgroup)	Lower body mass index (per 1 kg/m^2^ decrease), OR (95% CI)	*P* for interaction	Lower serum albumin (per 1 g/L decrease), OR (95% CI)	*P* for interaction	Higher procalcitonin (per 1 ng/ml increase), OR (95% CI)	*P* for interaction
Age	
<65 years (*N* = 93; malnutrition = 20, 21.5%)	1.64 (1.22–2.22)	0.723	1.15 (1.03–1.28)	0.827	1.44 (1.05–1.97)	0.914
≥65 years (*N* = 118; malnutrition = 39, 33.1%)	1.56 (1.23–1.96)		1.12 (1.03–1.22)		1.41 (1.05–1.90)	
Sex	
Male (*N* = 125; malnutrition = 31, 24.8%)	1.61 (1.27–2.08)	0.901	1.15 (1.05–1.25)	0.948	1.43 (1.06–1.93)	0.876
Female (*N* = 86; malnutrition = 28, 32.6%)	1.54 (1.18–2.04)		1.12 (1.02–1.25)		1.40 (1.01–1.95)	
COPD	
No (*N* = 151; malnutrition = 34, 22.5%)	1.52 (1.20–1.92)	0.284	1.11 (1.03–1.20)	0.213	1.39 (1.03–1.87)	0.742
Yes (*N* = 60; malnutrition = 25, 41.7%)	1.72 (1.25–2.38)		1.18 (1.04–1.32)		1.48 (1.05–2.08)	
History of malignancy	
No (*N* = 199; malnutrition = 56, 28.1%)	1.59 (1.27–1.96)	0.768	1.14 (1.05–1.22)	0.684	1.42 (1.09–1.86)	0.927
Yes (*N* = 12; malnutrition = 3, 25.0%)	1.69 (0.57–5.00)		1.20 (0.88–1.67)		1.51 (0.82–2.77)	
Disease severity (APACHE II)	
<18 (*N* = 115; malnutrition = 24, 20.9%)	1.52 (1.16–2.00)	0.441	1.11 (1.01–1.22)	0.386	1.45 (1.02–2.05)	0.808
≥18 (*N* = 96; malnutrition = 35, 36.5%)	1.64 (1.27–2.13)		1.16 (1.05–1.28)		1.39 (1.02–1.91)	

APACHE II, acute physiology and chronic health evaluation II; CI, confidence interval; COPD, chronic obstructive pulmonary disease; OR, odds ratio.

Stratified models were adjusted for the same covariates as the primary multivariable model except for the stratification variable.

P for interaction values were obtained by adding multiplicative interaction terms to the full model.

Continuous predictors were coded in the risk-increasing direction to improve clinical interpretability.

### 3.5 Association between malnutrition and clinical outcomes

Compared with patients without malnutrition, those with malnutrition had higher in-hospital mortality, higher 28-day mortality, and a higher rate of ICU admission. Sepsis or septic shock was also more frequent in the malnutrition group. In addition, patients with malnutrition had longer hospital length of stay, longer ICU length of stay among those admitted to the ICU, and higher hospitalization costs. Although invasive mechanical ventilation was more frequent in the malnutrition group, the between-group difference was not statistically significant. For secondary outcomes, acute kidney injury and multiple organ dysfunction syndrome were more frequent in the malnutrition group. In contrast, no statistically significant between-group differences were observed for acute respiratory distress syndrome, vasopressor use, continuous renal replacement therapy, or antibiotic escalation (Table 4).

**Table 4:** Clinical outcomes and treatment-related indicators by nutritional status.

Outcome/indicator	Malnutrition (*n* = 59)	Non-malnutrition (*n* = 152)	*P*-value
In-hospital mortality, *n* (%)	12 (20.3)	15 (9.9)	0.041
28-day mortality, *n* (%)	14 (23.7)	18 (11.8)	0.031
ICU admission, *n* (%)	36 (61.0)	68 (44.7)	0.034
Invasive mechanical ventilation, *n* (%)	18 (30.5)	29 (19.1)	0.073
Sepsis or septic shock, *n* (%)	27 (45.8)	46 (30.3)	0.034
Hospital length of stay, days, median [IQR]	15.7 [12.0, 22.6]	11.6 [8.7, 15.2]	<0.001
ICU length of stay, days, median [IQR]	6.8 [5.2, 9.6]	5.5 [3.7, 7.9]	0.031
Hospital costs, CNY, median [IQR]	77,223 [53,819, 105,370]	57,190 [41,486, 74,233]	<0.001
ARDS, *n* (%)	17 (28.8)	27 (17.8)	0.076
AKI, *n* (%)	15 (25.4)	20 (13.2)	0.032
MODS, *n* (%)	10 (16.9)	11 (7.2)	0.034
Vasopressor use, *n* (%)	24 (40.7)	41 (27.0)	0.053
CRRT use, *n* (%)	7 (11.9)	7 (4.6)	0.069
Antibiotic escalation, *n* (%)	21 (35.6)	38 (25.0)	0.124

ICU, intensive care unit; IQR, interquartile range; CNY, Chinese yuan; ARDS, acute respiratory distress syndrome; AKI, acute kidney injury; MODS, multiple organ dysfunction syndrome; CRRT, continuous renal replacement therapy. ICU length of stay was calculated among patients admitted to the ICU.

### 3.6 Univariable and multivariable analyses evaluating the impact of malnutrition on clinical outcomes

Univariable analyses showed that malnutrition was significantly associated with in-hospital mortality, 28-day mortality, ICU admission, an increased hazard of 28-day death, and a lower rate of discharge alive, while the corresponding association with invasive mechanical ventilation was not statistically significant. After adjustment for pre-specified covariates, malnutrition remained significantly associated with in-hospital mortality, 28-day mortality, ICU admission, invasive mechanical ventilation, an increased hazard of 28-day death, and a lower hazard of discharge alive (Table 5).

**Table 5:** Univariable and multivariable models evaluating the impact of malnutrition on prognosis.

Outcome/endpoint	Univariable effect (95% CI)	*P*-value	Multivariable effect (95% CI)	*P*-value
In-hospital mortality	OR 2.33 (1.02–5.34)	0.045	OR 2.43 (1.10–5.35)	0.028
28-day mortality	OR 2.32 (1.07–5.03)	0.034	OR 2.36 (1.12–4.98)	0.024
ICU admission	OR 1.93 (1.05–3.57)	0.035	OR 1.78 (1.01–3.15)	0.047
Invasive mechanical ventilation	OR 1.86 (0.94–3.70)	0.076	OR 1.85 (1.01–3.39)	0.046
28-day survival (event = death)	HR 2.21 (1.20–4.08)	0.011	HR 2.09 (1.11–3.93)	0.022
Time to discharge alive (event = discharge)	HR 0.66 (0.52–0.84)	0.001	HR 0.70 (0.54–0.91)	0.007

Univariable analyses were performed first to estimate the crude association between malnutrition and each outcome.

Multivariable logistic regression was used for binary outcomes, and multivariable Cox proportional hazards regression was used for time-to-event outcomes.

Multivariable models were adjusted for age, sex, APACHE II score, heart failure, and chronic kidney disease.

OR, odds ratio; HR, hazard ratio; CI, confidence interval.

For time to discharge alive, an HR <1 indicates a lower rate of discharge, corresponding to longer hospitalization.

### 3.7 Sensitivity analyses and robustness checks

To assess the robustness of the primary findings, we performed a series of sensitivity analyses addressing alternative GLIM phenotypic definitions, exclusion or inclusion of selected patient subgroups, missing-data handling, symptom-duration restriction, and a minimally adjusted model without APACHE II. Detailed results are provided in Supplementary Table S3. Overall, the associations between malnutrition and adverse outcomes remained directionally consistent across all sensitivity analyses. For 28-day mortality and time to discharge alive, the associations remained statistically significant in all scenarios examined. For ICU admission, the direction of association was preserved throughout, although statistical significance was attenuated in several alternative-definition and restricted-cohort analyses. These findings support the overall robustness of the primary results.

## 4 Discussion

Our study extends the current evidence base by applying a GLIM-based framework to a well-characterized cohort of patients with severe pneumonia and by evaluating both factors associated with malnutrition at admission and its prognostic associations after adjustment for major clinical confounders, including illness severity (20). In this cohort, malnutrition was common at presentation and was associated with a less favorable admission profile characterized by greater inflammatory burden, higher illness severity, and heavier comorbidity burden. Admission malnutrition was also associated with less favorable short-term outcomes, including higher mortality, greater need for intensive care, and delayed discharge, and these associations remained directionally robust across sensitivity analyses. Within the limits of this retrospective observational design, however, the present data support an association between GLIM-defined malnutrition and adverse outcomes rather than a direct causal role of malnutrition in poor prognosis. In severe pneumonia, GLIM-defined malnutrition may be more appropriately interpreted as a marker of overall clinical vulnerability and acute illness burden. In this context, standardized GLIM adjudication, together with supplementary characterization of phenotypic components and early nutritional management, may improve the interpretability of nutritional status in this population and assist in identifying patients who present with a higher-risk overall profile at admission (21).

A notable feature of this cohort is that GLIM classification was driven mainly by recent weight loss and low body mass index, whereas reduced muscle mass was documented less often because objective body-composition assessment was infrequently available in routine acute care (22, 23) This pattern is consistent with real-world practice in severe pneumonia, where nutritional evaluation at admission often depends primarily on pragmatic bedside indicators rather than formal body-composition methods. In multivariable analyses, lower body mass index, lower serum albumin, higher procalcitonin, higher APACHE II score, heart failure, and chronic kidney disease remained independently associated with malnutrition (24, 25). These findings indicate that the GLIM-defined nutritional phenotype identified in this setting clustered with both nutritional and non-nutritional features of vulnerability. At the same time, because several of these components overlap with systemic inflammation and illness severity, the present data do not allow a clear distinction between malnutrition as an independent biological process and malnutrition as a parallel reflection of acute severe illness (26). The subgroup analyses provide additional support for the stability of this pattern, as the associations of lower body mass index, lower serum albumin, and higher procalcitonin with malnutrition were directionally consistent across major clinical strata and showed no statistically significant interaction effects (27). Taken together, these results suggest that GLIM-based assessment may complement, rather than replace, conventional severity evaluation in hospitalized patients with severe pneumonia. (28, 29).

The prognostic analyses further showed that admission malnutrition was associated with less favorable short-term outcomes in severe pneumonia (30, 31). Patients classified as malnourished had higher in-hospital and 28-day mortality, greater ICU utilization, and longer hospitalization, and these associations remained statistically significant for mortality-related endpoints, ICU admission, invasive mechanical ventilation, and time to discharge alive after adjustment for major clinical confounders (32). These findings suggest that malnutrition may provide prognostically relevant information beyond standard severity assessment, while still being interpreted primarily as a marker of overall clinical vulnerability rather than as a confirmed causal determinant of poor prognosis (33, 34). An additional observation of practical relevance is that malnourished patients were more likely to receive formal nutritional support early after admission, yet documented energy and protein adequacy remained suboptimal in both groups (32, 35) This pattern suggests that recognition of nutritional risk in routine care did not necessarily correspond to adequate nutritional delivery during the acute phase of illness (36, 37). Accordingly, the present results support the value of structured nutritional screening and standardized GLIM-based assessment for risk recognition in severe pneumonia, but they do not establish that correction of malnutrition itself would necessarily improve outcomes. The consistency of the main associations across sensitivity analyses further supports the interpretability of these observations.

Our findings are broadly concordant with previous studies showing that GLIM-defined malnutrition and related nutritional phenotypes are associated with adverse outcomes in pneumonia. Asai et al. (38) reported that severe GLIM-defined malnutrition was associated with poorer long-term survival in community- onset pneumonia, whereas Shimizu et al. (39) found that severe malnutrition classified by GLIM body mass index cutoffs was associated with higher 30-day in-hospital mortality and prolonged length of stay, particularly in older adults. Murnane et al. (40) further showed that GLIM-defined malnutrition, especially when supported by CT-derived low muscle mass, identified more patients at nutritional risk than malnutrition defined by the International Classification of Diseases, Tenth Revision (ICD-10), and was more closely associated with clinically relevant pulmonary complications. Extending this literature, our study focused specifically on severe pneumonia, applied the full GLIM framework rather than a BMI-dominant classification alone, and jointly evaluated both determinants of malnutrition and its short-term prognostic associations within the same cohort. Taken together, the available evidence suggests that GLIM-defined malnutrition is a clinically meaningful correlate of vulnerability and outcome risk in pneumonia, while the extent to which it represents an independent biological contributor, rather than a parallel reflection of acute illness severity, remains uncertain.

Several limitations should be considered. First, because this was a retrospective single-center study, causality cannot be inferred, and residual confounding and selection bias cannot be excluded. Second, the operational definition of malnutrition in this acute setting overlapped to some extent with inflammation and illness severity, which requires cautious interpretation of the observed associations. Third, some nutrition-related markers, particularly serum albumin, may be influenced by acute-phase responses and fluid balance and therefore should not be interpreted as purely nutritional measures during severe infection. Fourth, objective body-composition assessment was not routinely available, limiting more precise characterization of reduced muscle mass within the GLIM framework. In addition, quantitative data on delivered energy and protein were incomplete and were therefore summarized descriptively rather than incorporated into the primary multivariable models. Finally, the number of events and the specific case mix may have affected model stability and generalizability. Accordingly, the present findings should be interpreted as evidence of association rather than causation, and prospective multicenter studies with standardized nutritional and body-composition assessment are needed for further validation.

## 5 Conclusion

Among patients with severe pneumonia, malnutrition was independently associated with lower body mass index and serum albumin, higher procalcitonin levels, higher APACHE II scores, and comorbid heart failure and chronic kidney disease. Malnutrition might also serve as a marker of worse short-term prognosis, as it was associated with higher mortality, greater intensive care unit utilization, more frequent organ dysfunction, prolonged hospital and intensive care unit stays, and higher hospitalization costs. These associations remained generally consistent across pre-specified subgroup and sensitivity analyses. Early structured nutritional assessment could therefore help improve risk stratification in patients with severe pneumonia.

## Data Availability

The original contributions presented in the study are included in the article/Supplementary material, further inquiries can be directed to the corresponding author.
