# Supplementary material for: Risk factors for malnutrition and its impact on prognosis in patients with severe pneumonia
Source: Front Nutr. 2026 Jun 10;13:1787876. doi: 10.3389/fnut.2026.1787876 (PMC13290660; doi:10.3389/fnut.2026.1787876)
Supplement: Supplementary file 1 [file Table_1.docx]

Supplementary Table S1. Distribution of GLIM phenotypic criteria and availability of body-composition assessment

| Section | Item | n/N (%) |
| --- | --- | --- |
| A. Frequency of individual phenotypic criteria among malnourished patients | Malnourished patients | 59/59 (100) |
|  | Low BMI | 37/59 (62.7) |
|  | Recent non-volitional weight loss | 44/59 (74.6) |
|  | Reduced muscle mass | 12/59 (20.3) |
| B. Overlap of phenotypic criteria among malnourished patients | Only low BMI | 12/59 (20.3) |
|  | Only recent weight loss | 16/59 (27.1) |
|  | Only reduced muscle mass | 1/59 (1.7) |
|  | Low BMI + recent weight loss | 19/59 (32.2) |
|  | Low BMI + reduced muscle mass | 2/59 (3.4) |
|  | Recent weight loss + reduced muscle mass | 5/59 (8.5) |
|  | Low BMI + recent weight loss + reduced muscle mass | 4/59 (6.8) |
| C. Availability of objective body-composition methods | Any objective body-composition method available, full cohort | 26/211 (12.3) |
|  | Any objective body-composition method available, malnutrition group | 14/59 (23.7) |
|  | BIA available, full cohort | 8/211 (3.8) |
|  | BIA available, malnutrition group | 4/59 (6.8) |
|  | CT-based assessment available, full cohort | 13/211 (6.2) |
|  | CT-based assessment available, malnutrition group | 8/59 (13.6) |
|  | Ultrasound-based assessment available, full cohort | 5/211 (2.4) |
|  | Ultrasound-based assessment available, malnutrition group | 2/59 (3.4) |
|  | Reduced muscle mass confirmed among objectively assessed malnourished patients | 12/14 (85.7) |

Abbreviations: GLIM, Global Leadership Initiative on Malnutrition; BMI, body mass index; BIA, bioelectrical impedance analysis; CT, computed tomography.

Phenotypic criteria in Section A are not mutually exclusive; therefore, percentages exceed 100%. Categories in Section B are mutually exclusive and sum to 100% of malnourished patients. In Section C, each patient was classified according to the first or primary objective body-composition method available in the medical record.

Supplementary Table S2. Early nutritional management during the first 72 h of hospitalization by nutritional status

| Variable | Malnutrition (n=59) | Non-malnutrition (n=152) | Total (n=211) | P value |
| --- | --- | --- | --- | --- |
| Any formal nutrition support within first 72 h, n (%) | 48 (81.4) | 96 (63.2) | 144 (68.2) | 0.011 |
| Primary early nutrition route among those receiving support, n (% of supported patients) |  |  |  | 0.413 |
| — Oral diet/ONS | 8 (16.7) | 28 (29.2) | 36 (25.0) |  |
| — EN | 28 (58.3) | 45 (46.9) | 73 (50.7) |  |
| — PN | 5 (10.4) | 9 (9.4) | 14 (9.7) |  |
| — EN + PN | 7 (14.6) | 14 (14.6) | 21 (14.6) |  |
| Time to first nutrition support, h, median [IQR] | 28 [18, 41] | 31 [20, 45] | 30 [19, 44] | NR |
| Nutrition support initiated within 48 h, n (% of total group) | 39 (66.1) | 93 (61.2) | 132 (62.6) | 0.508 |
| Documented energy delivery available, n | 43 | 87 | 130 | 0.036 |
| Mean delivered energy during first 72 h, kcal/kg/day, median [IQR] | 13.4 [10.8, 16.1] | 14.2 [11.5, 17.0] | 13.8 [11.1, 16.7] | NR |
| Energy adequacy ≥70% of estimated target by 72 h, n/N (%) | 18/43 (41.9) | 38/87 (43.7) | 56/130 (43.1) | 0.844 |
| Documented protein delivery available, n | 43 | 87 | 130 | 0.036 |
| Mean delivered protein during first 72 h, g/kg/day, median [IQR] | 0.62 [0.45, 0.81] | 0.68 [0.50, 0.87] | 0.66 [0.48, 0.85] | NR |
| Protein adequacy ≥70% of 1.3 g/kg/day target by 72 h, n/N (%) | 10/43 (23.3) | 24/87 (27.6) | 34/130 (26.2) | 0.597 |

Abbreviations: ONS, oral nutritional supplements; EN, enteral nutrition; PN, parenteral nutrition; IQR, interquartile range; NR, not reported. Time to first nutrition support was calculated among patients who received formal nutrition support within the first 72 h.

Supplementary Table S3. Sensitivity analyses for the independent prognostic impact of malnutrition

| Analysis scenario | Malnutrition definition / restriction | Outcome | Effect estimate (95% CI) | P value |
| --- | --- | --- | --- | --- |
| Primary analysis | GLIM (primary definition), N=211 (59 malnourished) | 28-day mortality | OR 2.36 (1.12–4.98) | 0.024 |
|  |  | ICU admission | OR 1.78 (1.01–3.15) | 0.047 |
|  |  | Time to discharge alive | HR 0.70 (0.54–0.91) | 0.007 |
| Sensitivity 6.1A | GLIM with stricter phenotypic thresholds, N=211 (48 malnourished) | 28-day mortality | OR 2.21 (1.01–4.82) | 0.044 |
|  |  | ICU admission | OR 1.73 (0.95–3.14) | 0.072 |
|  |  | Time to discharge alive | HR 0.72 (0.55–0.94) | 0.015 |
| Sensitivity 6.1B | GLIM with adjusted BMI cutoffs (broader capture), N=211 (66 malnourished) | 28-day mortality | OR 2.18 (1.09–4.38) | 0.027 |
|  |  | ICU admission | OR 1.69 (0.98–2.91) | 0.059 |
|  |  | Time to discharge alive | HR 0.71 (0.56–0.90) | 0.006 |
| Sensitivity 6.2 | Excluding malignancy (n=12) and advanced cirrhosis (n=9), N=190 | 28-day mortality | OR 2.29 (1.04–5.05) | 0.039 |
|  |  | ICU admission | OR 1.74 (0.97–3.12) | 0.063 |
|  |  | Time to discharge alive | HR 0.72 (0.55–0.94) | 0.015 |
| Sensitivity 6.3 | Missing-data strategy: multiple imputation (20 datasets) | 28-day mortality | OR 2.33 (1.10–4.93) | 0.026 |
|  |  | ICU admission | OR 1.76 (1.00–3.11) | 0.049 |
|  |  | Time to discharge alive | HR 0.71 (0.55–0.92) | 0.01 |
| Sensitivity 6.4 | Excluding patients with symptom duration >14 days before admission, N=188 (50 malnourished) | 28-day mortality | OR 2.41 (1.08–5.39) | 0.031 |
|  |  | ICU admission | OR 1.83 (1.00–3.34) | 0.049 |
|  |  | Time to discharge alive | HR 0.69 (0.52–0.91) | 0.009 |
| Sensitivity 6.5 | Including previously excluded patients with terminal malignancy or overt cachexia, N=217 (65 malnourished) | 28-day mortality | OR 2.52 (1.23–5.15) | 0.012 |
|  |  | ICU admission | OR 1.81 (1.04–3.15) | 0.036 |
|  |  | Time to discharge alive | HR 0.68 (0.53–0.88) | 0.003 |
| Sensitivity 6.6 | Minimally adjusted model without APACHE II: age, sex, heart failure, and chronic kidney disease | 28-day mortality | OR 2.88 (1.39–5.98) | 0.004 |
|  |  | ICU admission | OR 2.07 (1.19–3.60) | 0.01 |
|  |  | Time to discharge alive | HR 0.64 (0.50–0.83) | 0.001 |

Abbreviations: OR, odds ratio; HR, hazard ratio; CI, confidence interval; GLIM, Global Leadership Initiative on Malnutrition; APACHE II, Acute Physiology and Chronic Health Evaluation II; ICU, intensive care unit. For time to discharge alive, an HR <1 indicates a lower discharge rate, corresponding to longer hospitalization.

Primary analysis and Sensitivities 6.1A–6.5 were adjusted for age, sex, APACHE II score, heart failure, and chronic kidney disease. Sensitivity 6.6 was adjusted for age, sex, heart failure, and chronic kidney disease only.
